# An Unsupervised Learning Algorithm for the Automatic Classification of Coronary Artery Lesions

**DOI:** 10.7759/cureus.88638

**Published:** 2025-07-24

**Authors:** Julia Szopinska, Piotr A Regulski, Maciej Mazurek, Marcin Grabowski, Piotr Wendykier, Rafal Jozwiak

**Affiliations:** 1 Department of Dental and Maxillofacial Radiology, Laboratory of Digital Imaging and Virtual Reality, Medical University of Warsaw, Warsaw, POL; 2 Department of Cardiology, Medical University of Warsaw, Warsaw, POL; 3 Innovation Centre for Digital Medicine, National Information Processing Institute, Warsaw, POL

**Keywords:** atherosclerotic plaque classification, clustering algorithms, computed tomography, coronary artery disease, vessel segmentation

## Abstract

Background

Coronary artery disease (CAD) remains a leading cause of mortality globally. Accurate identification and characterization of significant coronary artery lesions via CT is important for proper diagnosis and patient management. However, current supervised techniques for lesion identification require detailed manual annotations, which are both labor-intensive and prone to human error. While semi-supervised or weakly supervised approaches can partially reduce manual annotation burden, they still depend on annotated datasets, which remain limited and inconsistent in CAD research. Therefore, there is a strong clinical motivation for developing robust unsupervised methods that eliminate annotation dependency.

Aim

This study aims to develop and evaluate a novel unsupervised method utilizing clustering algorithms to automatically classify and characterize significant lesions in coronary arteries from CT images, thereby addressing limitations of supervised and semi-supervised methods.

Methods

We analyzed 45 anonymized coronary artery CT scans from patients hospitalized between 2018 and 2022, selected based on the presence of plaques causing at least 30% stenosis. Although small, the dataset represented a clinically relevant population with a diverse range of lesion types (calcified, mixed, and soft plaques). Vessel segmentation was performed using nnU-Net, followed by skeletonization and extraction of statistical and Haralick texture features. Dimensionality reduction was executed using principal component analysis, and lesion clustering was conducted using both k-means and a hybrid clustering algorithm. Supervised methods are defined as algorithms that require labeled data for training, whereas unsupervised methods, as applied in this study, do not require labeled data and instead rely solely on inherent patterns within the imaging features. The effectiveness of lesion classification, including calcified, mixed, and soft plaques, was assessed. Additionally, hemodynamic significance was verified by comparison with fractional flow reserve (FFR) measurements.

Results

Vessel segmentation yielded a mean Dice coefficient of 0.93, indicating high segmentation accuracy. The hybrid clustering algorithm demonstrated superior lesion classification performance, achieving sensitivity rates of 95.6% for calcified plaques, 88.3% for mixed plaques, and 74.1% for soft plaques. These performance indicators compare favorably to previously reported supervised and unsupervised approaches. Furthermore, the method reliably identified hemodynamically significant lesions as confirmed by FFR (n = 15 lesions).

Conclusions

Our proposed unsupervised clustering-based method effectively classifies and characterizes coronary artery lesions without the need for manual annotations. However, the small sample size and limited number of lesions validated by FFR (n = 15) restrict broad generalizations and clinical translation. External validation on larger, multicenter datasets is essential to confirm these promising findings. This method offers a practical, accurate, and efficient diagnostic approach, potentially streamlining clinical workflow and improving patient outcomes.

## Introduction

Coronary artery disease (CAD) is a major clinical condition that is associated with poor quality of life and unfavorable prognoses. In developed countries, CAD is one of the leading causes of mortality [1]. Accurately and effectively detecting significant stenoses in coronary arteries is essential for diagnosing CAD, as doing so enables the early identification of changes that may require interventional treatments. Ongoing advancements in image processing continue to focus on improving and automating the diagnostic process.

The existing methods for extracting and classifying lesions are mainly based on supervised algorithms [2,3]. Convolutional neural networks are the most commonly used segmentation approaches, whereas support vector machines and random forests are frequently employed for classification tasks [2,3]. However, these methods require extensive manual annotations, which are labor-intensive, costly, and notably affected by interobserver variability. This variability arises due to subjective interpretations of lesion boundaries and the limited resolution of CT imaging, resulting in blurred and unclear plaque borders [4,5]. The inherent inconsistencies and resource demands associated with manual delineation limit the scalability and reliability of supervised approaches, motivating exploration into robust unsupervised techniques. The intensive and costly process of annotating lesions further diminishes the scalability and consistency of supervised methods that rely on precise labeling. As a result, interest in unsupervised methods, which do not require exhaustive annotations and may additionally facilitate the identification and characterization of novel atherosclerotic plaque types, is increasing.

Unsupervised methods offer the potential to identify patterns and classifications without requiring explicit labels, thus addressing several of the limitations that are inherent in supervised learning. However, despite these advantages, unsupervised methods have historically been underutilized in medical imaging scenarios because of challenges related to interpretability, accuracy, and clinical applicability. For example, traditional clustering algorithms may not effectively account for the complex structural and functional variations within coronary vessels, leading to clusters that lack clinical relevance or robustness. Unlike previous unsupervised methods that mainly rely on basic statistical analyses of intensity data, our study introduces a novel unsupervised method leveraging advanced texture descriptors combined with vessel skeletonization and a two-stage hybrid clustering algorithm (k-means integrated with Ward’s hierarchical clustering). The clustering step involves grouping similar data points based on statistical metrics and Haralick texture features extracted from vessel images, enhancing the precision and clinical relevance of lesion classifications. Another key step in this process is skeletonization, which is a technique that reduces complex structures such as coronary arteries to their essential centerlines, providing a more manageable representation for analysis purposes while preserving the critical morphological information. Unlike purely unsupervised algorithms that struggle to balance computational feasibility with high precision, our two-stage clustering framework is specifically optimized for large-scale volumetric data, ensuring both accuracy and clinical interpretability. Compared to traditional supervised methods, the proposed solution does not depend on the explicit manual labeling of thousands of voxels or tedious plaque delineations. We hypothesize that an unsupervised hybrid clustering method integrating skeletonization and texture-based statistical features can accurately classify different types of coronary artery lesions (calcified, mixed, and soft plaques) and reliably identify hemodynamically significant stenoses.

During the diagnostic process, in addition to detecting and classifying lesions, it is important to differentiate those that are hemodynamically significant and may require interventional treatments. This is particularly important from a practical point of view, as CT scans exhibit high sensitivity in terms of detecting such changes (94%) but low specificity (approximately 40%) [6], leading many patients to undergo unnecessary invasive diagnostic procedures. While fractional flow reserve (FFR) modelling methods have been developed, challenges remain concerning the real-world data required for training and testing such models. The insertion of a catheter into a blood vessel for measuring pressure disrupts blood flow and adversely affects the accuracy of the obtained readings [7].

This study aimed to overcome these challenges by introducing a novel unsupervised framework that leverages advanced segmentation and clustering techniques tailored to coronary artery imaging. The objective was to achieve a detailed representation of vessel morphology and plaque characteristics through the incorporation of coronary artery skeletonization and a range of statistical descriptors. These features were then analyzed via k-means and hybrid clustering algorithms to assess both the diagnostic accuracy and clinical relevance of the proposed approach, facilitating a comprehensive characterization of coronary vessels that distinguished healthy arteries from those with calcified, mixed, and soft plaques. With rapid processing times, the proposed framework has practical potential for real-time integration into clinical workflows, enabling timely and accurate clinical decision-making, thereby reducing reliance on manual delineations and minimizing unnecessary invasive procedures.

## Materials and methods

The retrospective analysis was conducted on 45 anonymized CT scans of coronary arteries acquired with intravenous contrast enhancement from patients hospitalized at the First Department of Cardiology of the Medical University of Warsaw (Central Clinical Hospital, Banacha 1A) between 2018 and 2022. The inclusion criteria were age ≥18 years, undergoing coronary artery CT angiography with intravenous contrast enhancement, and confirmed presence of coronary artery plaques causing at least 30% stenosis, recognized clinically as indicative of significant CAD progression. Patients were excluded if they were younger than 18 years, had incomplete or suboptimal quality CT imaging data, exhibited coronary artery stenosis below 30%, or lacked sufficient clinical documentation describing plaque characteristics. The study sample included 26 men and 19 women, with ages ranging from 38 to 83 years (mean age = 65 years; SD = 11.5 years). This study was conducted in accordance with the set ethical standards, and it received approval from the Local Ethics Committee at the Medical University in Warsaw (no. AKBE/245/2022), with informed consent obtained from all participants prior to their inclusion in the research.

Patients were selected based on the presence of plaques causing at least 30% stenosis, with the dataset comprising 18 calcified plaques, 17 soft plaques, and 49 mixed plaques. The inclusion threshold of 30% stenosis was selected based on clinical considerations, as coronary artery narrowing at this level is recognized by cardiologists as clinically relevant and potentially indicative of early stages of disease progression [6]. All plaques within each scan were included in the analysis to capture a comprehensive representation of the plaque types and variations that were present in the patient population.

The CT scans were acquired via a Toshiba Aquilion ONE CT scanner (Canon Medical Systems, Japan) at a voltage of 135 kVp and a current intensity of 660 mA, with a slice thickness of 0.5 mm. The images were saved in DICOM format, with a median scan resolution of 512 × 512 × 560 and a median voxel size of 0.43 × 0.43 × 0.5 mm. Radiologists provided detailed reports for each scan, documenting the lesion type (calcified, soft, or mixed), lesion location (the specific coronary artery segment), and degree of vessel stenosis. To minimize the risk of errors and ensure the precise identification of stenosis locations, regions with atherosclerotic plaques were manually segmented (directly on the CT scans) by an experienced cardiologist on the VisNow platform [8,9]. To ensure reproducibility and minimize potential observer bias, these segmentations were independently reviewed and validated by a second expert (radiologist). Discrepancies identified during this review were discussed and resolved by consensus. Manual annotations and evaluations by raters were conducted independently and blinded to the algorithmic clustering outputs. For 15 patients, coronary angiography results, including FFR values, were also available, enabling the assessment of the hemodynamic significance of their lesions under conditions comparable to those observed in the CT dataset.

**Figure 1:**
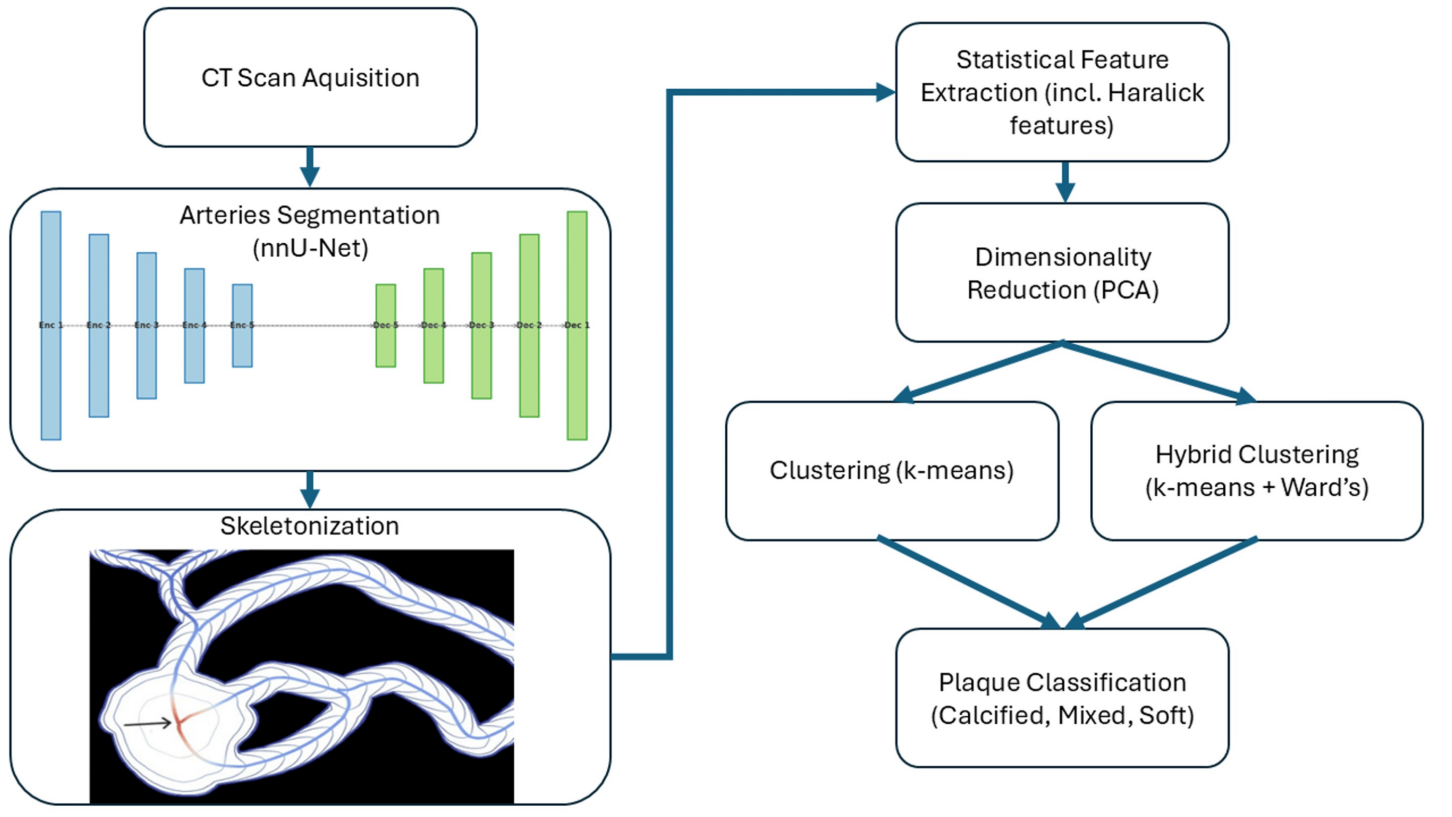
Proposed flow diagram for the unsupervised classification of coronary artery lesions. Steps include nnU-Net segmentation, skeletonization, feature extraction, PCA-based dimensionality reduction, and a hybrid clustering approach integrating k-means clustering and Ward’s hierarchical method. PCA, principal component analysis

The proposed algorithm for analyzing coronary artery lesions involves four key steps: vessel segmentation, skeletonization, statistical parameter computation, and clustering (Figure 1). The coronary vessels were segmented via the nnU-Net network [10], which was pretrained on 950 CT scans derived from the publicly available imageCAS dataset [11]. This dataset exhibits similar imaging characteristics to those of the study data, providing a suitable baseline for segmentation in this specific clinical context. The nnU-Net network was selected because of its state-of-the-art performance in biomedical image segmentation tasks, particularly its ability to adapt to different imaging modalities with minimal manual adjustments. The 3D full-resolution configuration was used, which typically employs a patch size of 96 × 160 × 160, a batch size of 12, and up to 320 feature maps in the deepest layers of the U-Net. The architecture included two convolutional layers per stage in both the encoder and decoder, with four pooling operations along each axis, reflecting the median post-transposition image shape of 275 × 512 × 512 voxels. The network used a CT-based normalization scheme to unify intensity distributions, and image data were resampled to the desired resolution using third-order interpolation, whereas segmentations relied on first-order interpolation to preserve the label integrity. The segmentation process was optimized with a combined Dice and cross-entropy loss function; stochastic gradient descent was implemented with an initial learning rate of 0.01, which was progressively reduced according to a polynomial decay schedule [4].

Several preliminary experiments were performed on a subset of training data to identify an optimal learning rate and loss function, evaluating multiple initial learning rates (0.001, 0.005, 0.01) and comparing polynomial decay with step-wise decay. Various loss functions were also tested (including Dice alone, cross-entropy alone, and focal-loss variants), and a combined Dice + cross-entropy approach was ultimately selected to balance overall volumetric accuracy with a precise boundary delineation. This strategy aligns with recommended nnU-Net settings, which have been validated across a range of biomedical segmentation tasks. The segmentation results were reviewed and validated by a radiologist, confirming their adequacy for subsequent steps of the algorithm.

The segmented blood vessels were skeletonized using a distance transform-based algorithm implemented via the VisNow platform [8,9], which was specifically designed for three-dimensional images. We selected the distance transform-based skeletonization approach due to its robustness, ease of integration with binary vessel segmentations, and straightforward computational requirements for three-dimensional datasets. Graph-based methods, although capable of tracking vascular structures, typically demand intricate graph construction steps and extensive parameter tuning, which can complicate large-scale analyses. Deep learning-based skeletonization approaches often require sizeably labeled datasets and substantial training procedures, making them less feasible in scenarios where ground-truth skeleton annotations are unavailable [12]. The algorithm begins by identifying central points within vessel structures via a weighted distance map, and it generates a distance map from these central points to each voxel in the artery. The local maxima among the distance values and vector angles between neighboring voxels are used to determine the endpoints and saddle points. These points are then connected to form a skeleton, with any segments shorter than a preset length threshold removed to yield a streamlined centerline representation of each artery. This skeletonization approach was chosen for its robustness to noise and its compatibility with the binary nature of segmented images.

Statistical metrics were computed along the vessel skeleton by analyzing it in a segment-by-segment manner. For each segment, perpendicular vectors were calculated to establish a local coordinate system, allowing the vessel to be analyzed from edge to edge and along its centerline. Metrics were calculated within cubic subregions (3 × 3 × 3 voxel windows) and included the mean, median, first and third quartiles, SD, kurtosis, skewness, and gradients along three axes. Additionally, Haralick texture features, including contrast, homogeneity, dissimilarity, entropy, difference entropy, sum entropy, energy, correlations, and the maximum probability, were calculated, capturing both the structural and textural variations that were relevant to plaque characterization.

The CT pixel values, represented on the Hounsfield scale from -1000 to 3000, were quantized into 32 equal intensity levels (each with a width of approximately 125 HU) to reduce the imposed memory demands and prevent the co-occurrence matrices used for the Haralick features from being sensitive to minor pixel variations. The primary components emphasized texture features, gradient directions, and intensity levels, which were identified as essential for distinguishing plaque types.

The clustering analysis employed both the k-means algorithm and a hybrid approach that integrates k-means with Ward’s hierarchical algorithm to achieve increased clustering precision. The hybrid algorithm was specifically designed to address the computational limitations of hierarchical clustering when addressing large datasets, such as the approximately 200,000 voxels per reconstructed coronary artery in this study. Pure hierarchical clustering, while effective for high-precision grouping, is computationally demanding and becomes impractical at such data scales due to its quadratic temporal complexity. In the hybrid approach, k-means clustering is initially used to partition the input dataset into preliminary clusters, reducing the dimensionality of the dataset and the imposed computational load. Subsequently, Ward’s hierarchical clustering scheme refines these clusters by minimizing the within-cluster variance, leveraging the output of k-means to generate more structurally coherent and clinically meaningful clusters. Ward’s algorithm iteratively merges smaller clusters that are most similar in terms of within-cluster variance. This step is particularly effective at identifying subtle differences in plaque morphology and density, thereby enhancing the classification accuracy for the more challenging soft and mixed plaques. This two-step approach provides the sensitivity that is necessary for detecting subtle plaque structure and texture variations across different vessel regions, offering a balance between computational efficiency and clustering accuracy.

The effectiveness of the proposed method was evaluated in terms of the segmentation accuracy of the plaques, the plaque classification results, and the ability to detect hemodynamically significant lesions. Quantitative performance metrics, including sensitivity, specificity, precision, and the F1 score, were employed to assess the accuracy of the plaque detection and classification results and were calculated at the plaque level, treating each individual lesion as a separate unit of analysis. These metrics were calculated separately for each type of stenosis - calcified, soft, and mixed - to provide a detailed understanding of the performance achieved by the proposed method across different plaque characteristics.

To evaluate the ability of the method to detect hemodynamically significant changes, a comparative analysis was conducted using FFR measurements obtained through coronary angiography performed for a subset of 15 patients. Lesions with FFR values that were ≤ 0.80 were classified as hemodynamically significant, requiring revascularization. This approach enabled a validation of the ability of the developed algorithm to detect and classify lesions with functional significance during coronary circulation, ensuring alignment with the clinically established hemodynamic criteria.

Statistical analyses were conducted to compare the performance of the k-means and hybrid algorithms. Paired t tests were used to compare the mean performance metrics (F1 scores) attained by the two algorithms for each plaque type. P values are reported to indicate the statistical significance of the differences observed between the algorithms, with p < 0.05 considered statistically significant. To quantify the precision of these performance comparisons, we also computed 95% CIs for the differences between algorithms.

## Results

**Figure 2:**
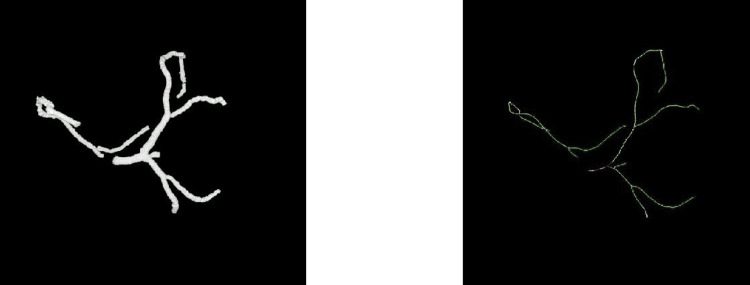
Segmented coronary artery (left) and coronary artery skeletons (right).

The coronary vessel segmentation approach achieved a mean Dice similarity coefficient of 0.93, indicating high accuracy and consistency. The nnU-Net segmentation model demonstrated 99% precision and 98% recall. Following segmentation, skeletonization was successfully applied, yielding accurate centerlines. The segmentation and skeletonization process is shown in Figure 2.

The optimal number of clusters was determined systematically using the elbow method. Specifically, we analyzed clustering performance for a range of two to 10 clusters. The elbow method indicated minimal additional reduction in within-cluster variance in five clusters, indicating optimal cluster cohesion and separation. The k-means and hybrid clustering algorithms identified five distinct clusters, with color coding provided based on the vessel widths and plaque types. Wide artery segments are marked in green, transitioning through yellow and orange for narrower segments and ending in red for the narrowest portions. Calcified plaques, which exhibit high radiological density, were isolated in a distinct blue cluster; soft plaques were presented in orange; and mixed plaques were described with a mix of blue, orange, and green. This color scheme visually distinguished different vessel segments according to their plaque types and degrees of narrowing.

**Figure 3:**
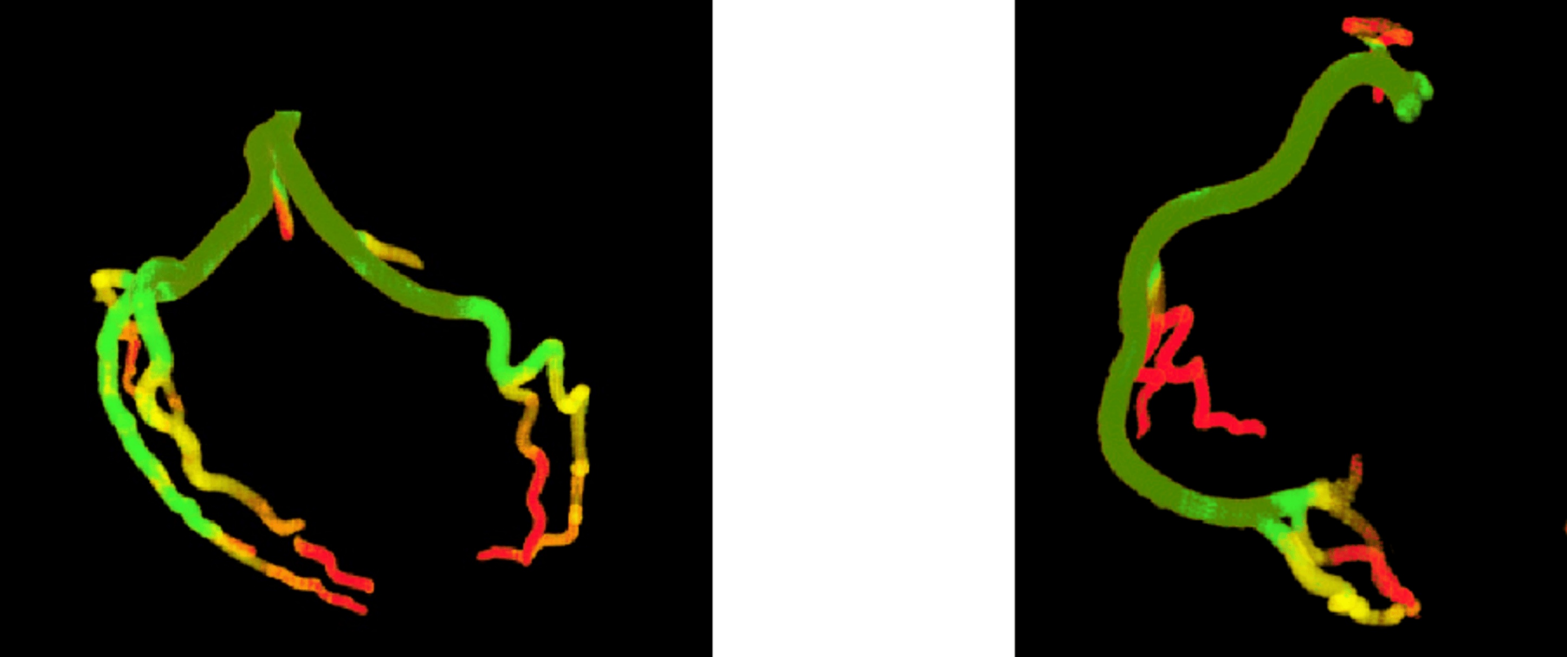
Clustering results produced for the coronary arteries without pathological changes: left coronary artery (left) and right coronary artery (right).

For vessels with no detectable lesions, the clustering process classified artery segments according to natural width variations. As shown in Figure 3, the arteries narrow progressively from the proximal end to the distal end. Healthy vessels were classified without misinterpretations, demonstrating that the clustering algorithm preserved anatomical fidelity in the nonpathological segments. No plaque areas were detected in the healthy arteries.

**Figure 4:**
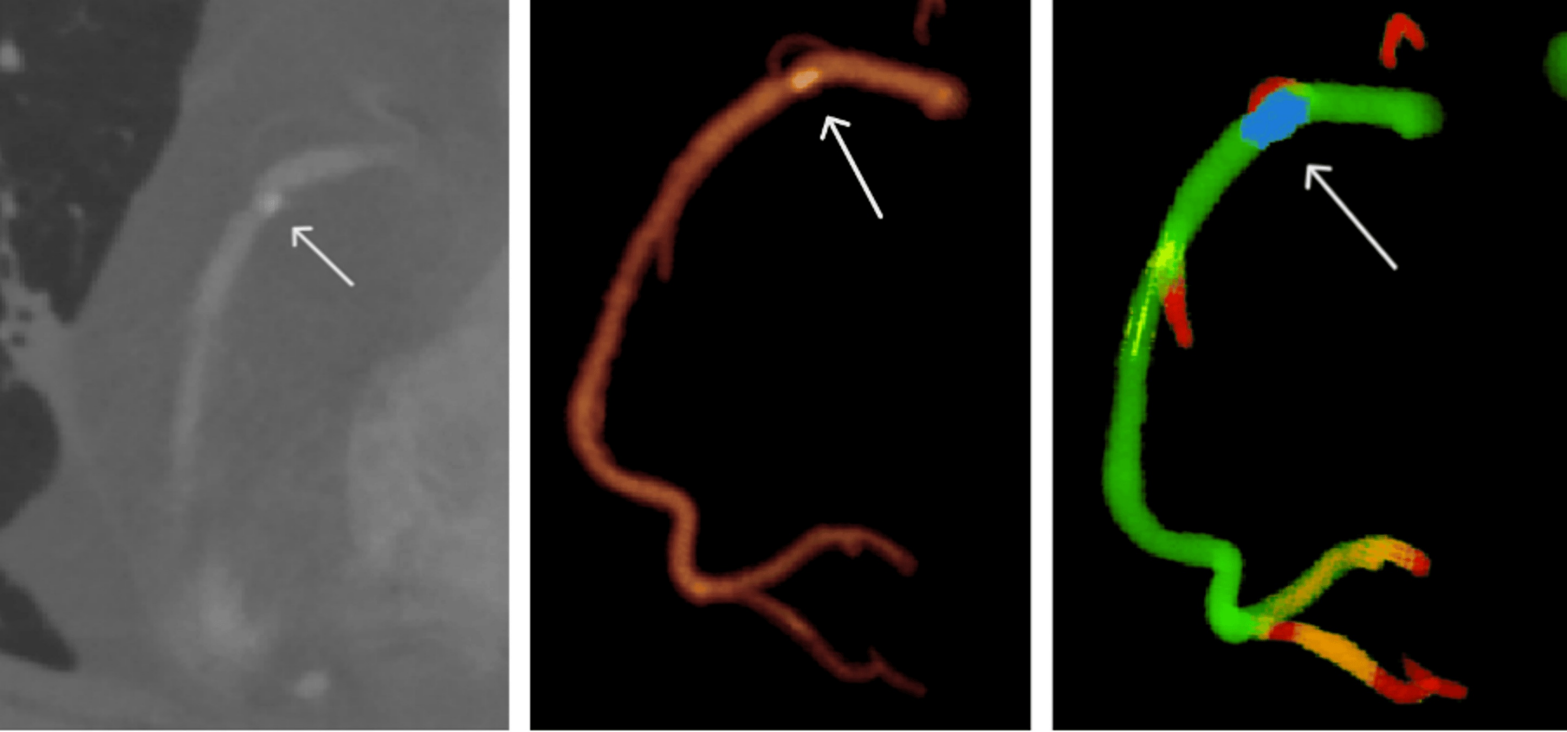
Right coronary artery with calcified plaque in the proximal segment. (left) CT image showing the calcified plaque (white arrow) with high attenuation values. (middle) 3D reconstruction illustrating the coronary artery’s overall shape and vessel width. (right) Clustering analysis results. The calcified plaque is isolated into a distinct blue cluster, reflecting its markedly higher HUs. The color scale transitions from green (wider, healthy segments) to red (narrower segments), highlighting how calcification coincides with a reduction in the artery area.

Owing to their high radiological density, the calcified plaques were accurately segmented and clustered into the blue category, as shown in Figure 4. The high specificity (99%) and sensitivity (94-95%) achieved for calcified plaque detection across both algorithms indicate their robust ability to identify and isolate calcified regions. The algorithmically segmented calcified regions showed a high degree of alignment with the manual segmentations produced by specialists. However, in some cases, the clustering algorithms extended the segmented areas to include adjacent regions. This effect was likely caused by disturbances from the contrast agent affecting the blood flow near the plaque, resulting in segmentation beyond the actual plaque boundaries [8]. Specifically, for the k-means algorithm, this deviation reached a radius of 2.3 ± 0.4 mm from the plaque, as measured by the Hausdorff distance (HD), whereas for the hybrid algorithm, the radius was slightly smaller, at 2.1 ± 0.3 mm HD. Compared with the k-means algorithm, the hybrid algorithm achieved significantly better segmentation performance in terms of the F1 score (p = 0.011), indicating more accurately delineated calcified plaques. Specifically, the hybrid algorithm yielded an F1 score of 95.9 ± 1.6%, surpassing the F1 score of 94.7 ± 1.8% produced by the k-means algorithm. These metrics collectively indicate that the hybrid method provided more precise and consistent segmentation results for calcified regions, aligning more accurately with the manual delineations of the experts.

**Figure 5:**
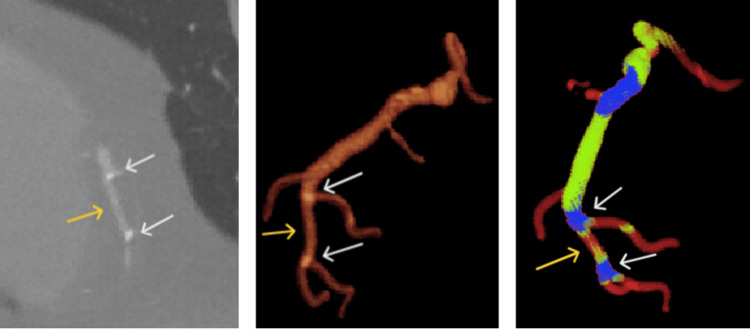
Right coronary artery with mixed etiology plaque; the white arrows indicate calcified components, whereas the yellow arrows indicate the soft components of mixed plaques.  (left) Oblique CT slice of the plaque; the white arrows point to the calcified regions (high attenuation), and the yellow arrows point to the approximate location of the softer (lipid/fibrotic) components (lower attenuation). (middle)  The 3D volume rendering displays the geometry of the artery and the extent of the plaque. (right)  Image of the clustering analysis results; calcified regions appear in a blue cluster, while the softer plaque portions blend with narrower vessel segments (orange/red clusters).

Mixed plaques contain both calcified and soft (lipid/fibrotic) components. The clustering algorithms effectively separated these components, with calcified regions classified into blue clusters and soft regions classified into clusters associated with vessel narrowing (red). The sensitivity, specificity, and F1 score achieved by the k-means algorithm were 85%, 98%, and 86%, respectively, and those of the hybrid algorithm were 88%, 98%, and 87%, respectively. Figure 5 shows a mixed plaque with distinct clusters for its calcified and soft portions. The combination of intensity and texture features enabled the accurate characterization of these complex lesions.

**Figure 6:**
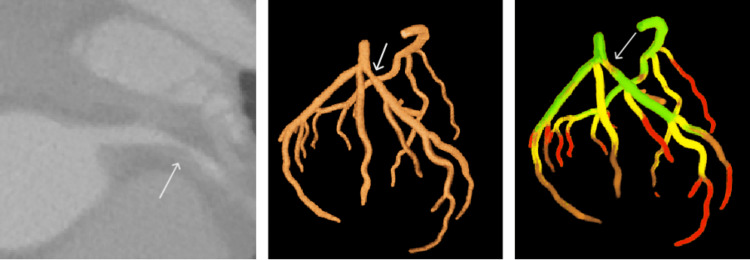
Left coronary artery with soft plaque in the ostium and the proximal segment of the LAD. (left) CT slice indicating a soft plaque (white arrow); (middle) 3D reconstruction of the coronary segment, emphasizing the plaque’s location (white arrow). (right) Clustering analysis, where the lower attenuation values of the plaque appear in the orange cluster (white arrow), indicating narrower, pathologic zones. LAD, left anterior descending artery

Soft plaques presented a greater challenge because of their lower contrast levels in CT images. The clustering algorithms identified these plaques as regions with reduced vessel diameters and altered texture features. The sensitivity, specificity, and F1 score achieved by the k-means algorithm were 71%, 95%, and 71%, respectively, and those of the hybrid algorithm were 74%, 96%, and 75%, respectively. In Figure 6, the area containing the soft plaque was classified into clusters representing narrower vessel segments (orange and red), where anatomically, a wider segment would be expected. Statistical measures such as lower mean HU values (mean HU: 100 ± 30) and increased entropy values were important for differentiating soft plaques from healthy tissue.

The hybrid algorithm demonstrated better performance across all plaque types, particularly for soft and mixed plaques (Table 1). In the statistical analysis of the produced F1 scores, the performances of the k-means and hybrid algorithms were compared for different plaque types via independent sample t tests. For calcified plaques, the t test yielded a t statistic of -3.33 with a p value of 0.0016 (95% CI: (93.0%, 96.3%) for the k-means algorithm and (94.5%, 97.3%) for the hybrid algorithm), indicating a statistically significant difference between the two algorithms. Similarly, for mixed plaques, the results were also statistically significant, with a t statistic of -4.72 and a p value of 0.0002 (95% CI: (83.1%, 88.2%) for the k-means algorithm and (85.4%, 91.8%) for the hybrid algorithm). In the soft plaque case, the t statistic was -3.74, and the p value was 0.0004 (95% CI: (80.1%, 82.5%) for the k-means algorithm and (82.6%, 85.3%) for the hybrid algorithm), further supporting the presence of a significant difference between the algorithms. When all the parameters obtained for all the plaque types were compared, the t test resulted in a t statistic of -1.85 and a p value of 0.0497.

**Table 1:** Algorithmic effectiveness for calcified, mixed, and soft plaques All reported values are expressed as percentages.

Plaque type	Algorithm	Sensitivity (%)	Specificity (%)	Precision (%)	F1 score (%)
Calcified	k-means	94.4 ± 2.1	99.2 ± 0.5	95.0 ± 2.0	94.7 ± 1.8
	Hybrid	95.6 ± 1.8	99.5 ± 0.3	96.2 ± 1.5	95.9 ± 1.6
Mixed	k-means	85.2 ± 3.5	97.8 ± 1.0	86.0 ± 3.2	85.6 ± 3.3
	Hybrid	88.3 ± 3.0	98.1 ± 0.8	88.9 ± 2.8	88.6 ± 2.9
Soft	k-means	70.6 ± 4.0	95.5 ± 1.5	72.0 ± 3.8	71.3 ± 3.9
	Hybrid	74.1 ± 3.8	95.9 ± 1.2	75.5 ± 3.6	74.8 ± 3.7

In the assessment of hemodynamically significant stenoses, the performance of the proposed method was compared with that of the FFR values derived from coronary angiography, and an FFR threshold of ≤ 0.80 was used to define significant lesions. The hybrid algorithm displayed strong accuracy, correctly identifying 12 of 15 significant cases, with 2 false negatives (FN) and 1 false positive (FP), thus indicating high sensitivity to clinically impactful stenoses. In contrast, the k-means algorithm correctly detected 11 of the 15 significant cases, resulting in 3 FNs and 1 FP.

While both algorithms effectively identified most of the hemodynamically significant plaques, the hybrid algorithm exhibited a slight advantage in terms of sensitivity, enhancing its potential clinical utility for detecting lesions that critically affect coronary blood flow.

## Discussion

This study presents a novel unsupervised method for the automatic characterization of significant stenoses in coronary vessels via clustering algorithms. By integrating an advanced segmentation process with nnU-Net, skeletonization, statistical feature extraction, and clustering analysis, we aimed to overcome the limitations associated with supervised methods, particularly the need for extensive and precise manual annotations.

The high accuracy achieved when detecting calcified plaques aligns with prior research that emphasized the reliability of CT imaging for identifying calcified lesions because of their distinct radiodensity [13]. However, soft plaques pose a greater challenge because their attenuation values are similar to those of the surrounding tissues [13]. The improved detection rates attained by our method for soft plaques suggest that the inclusion of texture features and a hybrid clustering approach can provide increased sensitivity, addressing a gap noted in earlier studies that relied solely on intensity-based methods [2]. The moderate sensitivity achieved for soft plaques (74.1%) highlights inherent challenges posed by subtle radiological contrast and diffuse plaque boundaries, making them particularly difficult to classify accurately. Despite these limitations, the observed classification performance can still meaningfully aid clinical decision-making by providing additional diagnostic confidence and potentially reducing unnecessary invasive diagnostic procedures. Even modest accuracy improvements (e.g., from 71% to 74% F1 score) can shift clinical decision thresholds, supporting more informed treatment decisions.

The results presented in our study are particularly noteworthy because of the unique focus on improving the sensitivity achieved when detecting soft coronary plaques via a hybrid clustering approach. While Jiang et al. [14], Hoshino et al. [15], and Zhang et al. [16] employed unsupervised learning techniques to analyze CAD characteristics, their objectives and approaches differed substantially from ours [17]. Jiang et al. [14] utilized unsupervised clustering to analyze the heterogeneity of clinical and plaque characteristics in patients with type 2 diabetes mellitus, offering insights into subgroup distinctions rather than directly detecting plaques. Hoshino et al. [15] focused on performing unsupervised hierarchical clustering to identify the coronary plaque subtypes associated with pericoronary inflammation, linking plaque phenotypes to adverse cardiac events. Zhang et al. [16] applied unsupervised clustering to select features for predicting ischemia-specific lesions, emphasizing the prediction of ischemia through feature selection rather than segmentation accuracy. In contrast, our study uniquely integrated texture analysis and a hybrid clustering algorithm, which directly enhanced the ability of the developed method to detect soft plaques.

The findings presented in this study stand in contrast with the literature, which has predominantly focused on supervised methods for detecting and segmenting coronary artery stenoses. Notably, supervised learning approaches require extensive labelled datasets, making them reliant on time-intensive and costly manual annotations. In the current literature, studies such as those of Zhang et al. [16], Kelm et al. [18], and Li et al. [19] reported that supervised segmentation models achieved high sensitivity and specificity values when detecting significant stenoses, particularly calcified plaques, with sensitivities reaching up to 94% and specificities of approximately 86% for specific plaque types. Despite their robust performance, these methods remain constrained by the quality and consistency of the input annotated data, often resulting in limited generalizability to heterogeneous patient populations.

More recent supervised studies, such as those of Freiman et al. [20], Kang et al. [21], and Hampe et al. [22], have focused on performing functional assessment via metrics such as the area under the curve (AUC) for FFR prediction. Earlier unsupervised work by Kang et al. [21] clustered raw HU intensities without texture or shape descriptors, leading to poor separation of lipid-rich tissue (F1 = 63%). Likewise, Hoshino et al. [15] used hierarchical clustering on pericoronary inflammation signatures, which do not capture intra-plaque texture, yielding low specificity (57%). Furthermore, hierarchical clustering in their work was applied for phenotypic classification associated with inflammation rather than direct plaque segmentation. By contrast, our hybrid scheme adds advanced texture analysis with Haralick features and skeleton-based morphometrics before hierarchical refinement, directly addressing those two shortcomings and boosting soft‑plaque F1 to 74.8%. While these models have shown promise, achieving AUCs of 0.78 to 0.8, they emphasize the detection of functionally significant stenoses rather than comprehensively classifying various types of plaques. Similarly, Zhou et al. [23] and Zreik et al. [24,25] achieved AUCs of 0.81 for artery-level detection and 0.87 at the patient level for functionally significant stenoses. However, the focus on patient-level metrics often obscures detailed plaque type classification, which is an area that is critical for clinical decision-making [26,27].

In contrast, our unsupervised approach uniquely addresses the tasks of segmenting and classifying both calcified and noncalcified plaques without the need for annotated training data. The hybrid clustering algorithm demonstrated competitive sensitivity (95.6%) and specificity (99.5%) for calcified plaques, aligning with the high specificities reported for supervised models in the literature while also extending its accurate detection capabilities to challenging soft plaques.

Additionally, Table 2 provides a broader comparison of the sensitivity, specificity, and F1 scores for supervised and unsupervised approaches across multiple studies, highlighting how our unsupervised hybrid clustering method achieves competitive or superior results for calcified, mixed, and soft plaques. Notably, while previous unsupervised methods such as those used by Kang et al. [21] and Hoshino et al. [15] showed lower specificity or F1 scores, supervised techniques [28-33] have reported similar sensitivity values in the range of 74-94% under various imaging protocols, but they typically require substantial manual annotations or rely on complex data labeling pipelines. In contrast, our unsupervised method sidesteps the need for extensive manual segmentation and still achieves strong classification performance - particularly for calcified plaques - while also exhibiting a reasonable balance for soft plaques. These findings emphasize the feasibility of an unsupervised pipeline that can match or exceed certain supervised baselines, thus reinforcing the clinical practicality of our proposed framework for coronary artery plaque detection and characterization.

**Table 2:** Comparison between supervised and unsupervised methods for the segmentation of stenosis by sensitivity. * For studies that reported only AUC, point sensitivity and F1 were approximated by (i) extracting the ROC coordinate closest to the Youden index when published or (ii) assuming class prevalence reported by the authors and inverting precision/recall formulae. These indirect estimates introduce ±3–5 pp uncertainty, which we explicitly acknowledge when comparing methods. AI-QCT, artificial intelligence-assisted quantitative coronary tomography; CNN, convolutional neural network; DAC-U-Net, dual-attention U-Net; FFR, fractional flow reserve (functional significance); SVM, support vector machine; TransCHD; hybrid CNN-transformer model; TSG; topological soft gradient

Study	Method	Sensitivity (%)	Specificity (%)	F1 score (%)
Proposed method	Unsupervised hybrid clustering	95.6 (calcified), 88.3 (mixed), 74.1 (soft)	99.5 (calcified), 98.1 (mixed), 95.9 (soft)	95.9 (calcified), 88.6 (mixed), 74.8 (soft)
Hoshino et al. [15]	Unsupervised clustering	64.7	57	32.4
Freiman et al. [20]	Supervised lumen segmentation	N/A	N/A	80.0*
Kang et al. [21]	Unsupervised clustering	93	81	63.2
Hampe et al. [22]	Supervised CNN + transformer (FFR)	N/A	N/A	78.0*
Zreik et al. [24]	Supervised CNN-RNN multi-task classification	N/A	N/A	78.5*
Zreik et al. [25]	Supervised CNN autoencoder + SVM (FFR)	N/A	N/A	81.0*
Hong et al. [27]	Supervised DAC-U-Net segmentation	N/A	N/A	79.2
Kang et al. [28]	Supervised lumen segmentation + vessel linearization	94	86	89.8*
Wang et al. [29]	Supervised level-set segmentation	74	93	82.4*
Wei et al. [30]	Supervised TSG + luminal analysis	93.5	N/A	N/A
Jonas et al. [31]	Supervised AI-QCT	80.9	N/A	N/A
Nannini et al. [32]	Supervised Cascaded 2.5D/3D U-Net	N/A	N/A	89.6
Zhao et al. [33]	Supervised hybrid CNN-transformer (TransCHD)	N/A	N/A	81.0*

In terms of detecting hemodynamically significant lesions, our method showed promise for improving the specificity over that of traditional CT angiography, which typically has low specificity (~40% for soft plaques) [6]. By integrating unsupervised clustering with a detailed statistical analysis, we provide a noninvasive means for better assessing the functional significance of detected lesions, potentially reducing the number of unnecessary invasive procedures performed.

The successful application of unsupervised clustering algorithms in this context has significant clinical implications. First, meaningful classification and characterization can be achieved for coronary atherosclerotic plaques without the extensive manual labeling required by supervised methods. This approach could substantially reduce the time and resources needed for model training, making advanced diagnostic tools more accessible in clinical settings. Given that the average processing time per case was 75 seconds (for the hybrid method) and 73 seconds (for the k-means methods), our proposed approach could be integrated into the clinical workflow for a near-real-time analysis, although further code optimizations or graphic-accelerated pipelines may be necessary to handle high-volume settings.

Second, the improved ability to detect hemodynamically significant stenoses suggests that our method could enhance patient stratification and decision-making processes. By more accurately identifying lesions that are functionally significant, clinicians can better determine which patients would benefit from revascularization techniques, such as percutaneous coronary intervention or coronary artery bypass grafting, thereby improving patient outcomes and optimizing the use of healthcare resources. Error analysis of false positives and false negatives in identifying hemodynamically significant lesions indicated that misclassification often occurred in mixed and soft plaques due to subtle intensity variations and blurred boundaries caused by contrast artifacts. Specifically, soft plaques with diffuse borders or mixed plaques containing significant soft components tended to be incorrectly clustered, highlighting inherent difficulties with these lesion types. These observations suggest that incorporating additional imaging features or refined preprocessing steps could potentially enhance the classification accuracy for these challenging plaque categories.

Because clustering assigns every voxel to one of five color-coded groups rendered on the 3D artery, interventional cardiologists can visually confirm that high-risk clusters (orange/red) align with luminal narrowing, while calcified clusters appear blue. During pilot readouts, all cardiologists reported the color map to be “intuitive,” supporting adoption.

Despite these encouraging results, several limitations must be acknowledged. First, the study was conducted on a relatively small cohort of 45 patients, with only 15 patients having corresponding FFR measurements available for assessing hemodynamic significance. This limited sample size might have impacted the generalizability of the findings and could cause the overinterpretation of the hemodynamic accuracy. Larger, multicenter studies are necessary to validate the effectiveness of the proposed method across more diverse populations. Additionally, class imbalance may bias clustering performance toward the more frequent plaque type (mixed plaques) and negatively affect the accuracy and generalizability for less represented types (particularly soft plaques). Future work will focus on expanding the dataset by collaborating with multiple clinical centers and including diverse patient demographics to address this concern. Conducting such multicenter studies will help verify the robustness of our approach, provide comprehensive representation across different plaque types and imaging conditions, and support broader clinical adoption. However, a notable advantage of this study is that the segmentation and clustering steps were conducted on distinct datasets. Specifically, vessel segmentation was performed using a model pretrained externally on the imageCAS dataset, whereas the clustering analysis was applied independently to our internal patient dataset. Despite these differing sources of data, the proposed unsupervised clustering approach demonstrated robust and clinically satisfactory performance, underscoring the method’s strong generalization potential and adaptability across datasets.

Second, while the hybrid algorithm attained improved detection rates for soft plaques, its sensitivity to these plaques remained lower than that achieved for calcified plaques. Soft plaques often present subtle imaging characteristics, making them more challenging to detect. An additional refinement of the feature extraction process could be beneficial for enhancing the sensitivity of the method to these harder-to-detect plaque types.

Third, the clustering algorithms occasionally delineated areas surrounding plaques that were larger than those marked by physicians, potentially highlighting regions where blood flow disturbances occurred. However, validating these peripheral regions poses a challenge because of the current limitations with respect to measuring blood flows at such fine scales. Future work incorporating computational fluid dynamics or advanced imaging techniques could provide insights into these surrounding areas and confirm the presence of disturbed flows. Finally, while the hybrid algorithm offered superior clustering performance, it required marginally longer computational times than those of the competing methods. In a clinical setting, computational efficiency is essential, and further optimization may be needed to ensure the practicality of the proposed method for routine use.

To address these limitations and build upon our findings, future research should prioritize several key areas. Expanding the dataset by increasing its sample size and incorporating data from multiple centers will be essential for increasing the robustness of the method and its generalizability across a broader patient population. Enhancing the feature extraction process also holds promise for attaining improved detection accuracy, particularly for soft plaques. The incorporation of additional features, such as higher-order texture measures or those derived from machine learning techniques, including deep learning-based feature extraction, could enable the method to capture more subtle plaque characteristics. This refinement may improve the sensitivity of the method to soft plaques, which often present imaging challenges because of their low contrast levels relative to those of the surrounding tissues. Finally, optimizing the computational performance of the proposed approach is crucial for its clinical implementation. Streamlining the algorithm to achieve faster processing times without compromising the resulting accuracy will ensure that the method remains feasible for routine use in clinical settings, where temporal efficiency is paramount.

## Conclusions

The developed unsupervised method has significant potential with respect to automatically classifying and characterizing significant coronary stenoses. Three types of changes were successfully characterized: calcified, mixed, and soft plaques. By significantly reducing reliance on extensive manual annotations, our approach addresses key limitations of supervised methods, particularly labor-intensive data labeling and susceptibility to interobserver variability. While our proposed method does not require a dedicated training set or explicit training procedures, larger and preferably multicenter datasets are necessary in future studies to robustly validate and generalize these findings to broader clinical practice. Nonetheless, this method has potential clinical implications, as improved noninvasive plaque characterization could facilitate earlier identification of hemodynamically significant lesions, enable better patient stratification for targeted interventions, and reduce unnecessary invasive diagnostic procedures. Future validation studies with larger, diverse, and external datasets are essential to confirm the clinical utility and scalability of our approach.
